# Patterns of Treatment Delay in Patients with Symptomatic Metastatic Epidural Spinal Cord Compression

**DOI:** 10.3390/cancers17040595

**Published:** 2025-02-10

**Authors:** Shilin Wang, James T. P. D. Hallinan, Cherie Lin Hui Tan, Khye Gin Eugene Chua, Alex Quok An Teo, Naresh Kumar, Gabriel Liu, Hwee Weng Dennis Hey, Joseph Thambiah, Leok-Lim Lau, Hee-Kit Wong, Yiong-Huak Chan, Jiong Hao Jonathan Tan

**Affiliations:** 1 Department of Orthopaedic Surgery, National University Hospital, National University Health System, Singapore 119074, Singapore; wsl.wangshilin@gmail.com (S.W.); alex_teo@nuhs.edu.sg (A.Q.A.T.); dosksn@nus.edu.sg (N.K.); gabriel_liu@nuhs.edu.sg (G.L.); doshhwd@nus.edu.sg (H.W.D.H.); dosjst@nus.edu.sg (J.T.); doslll@nus.edu.sg (L.-L.L.); doswhk@nus.edu.sg (H.-K.W.); 2 Department of Diagnostic Imaging, National University Hospital, National University Health System, Singapore 119074, Singapore; james_hallinan@nuhs.edu.sg; 3 Lee Kong Chian School of Medicine, Nanyang Technological University, Singapore 636921, Singapore; m220055@e.ntu.edu.sg (C.L.H.T.); m210071@e.ntu.edu.sg (K.G.E.C.); 4 Biostatistics Unit, Yong Loo Lin School of Medicine, National University of Singapore, Singapore 117597, Singapore; medcyh@nus.edu.sg

**Keywords:** spine, metastases, cord, compression, MESCC, treatment, delay, outcomes, function, independence

## Abstract

Background: Delays in the diagnosis and treatment of metastatic epidural spinal cord compression (MESCC) can potentially result in serious, deleterious effects on patient outcomes and postoperative morbidity. Delays may occur any at any step in the chain of care, increasing the risk of preventable complications. Objectives: This study, thus, aims to identify patterns of treatment delays and determine the predictive factors of postoperative ambulatory function in patients with symptomatic MESCC. Methods: Adult patients, aged >18 years, who underwent surgical treatment for MESCC between 2015 and 2022, were included for analysis in this retrospective study. Results: A total of 177 patients were included. The most significant delay contributing to total delay was patient delay (mean duration 41 days) followed by diagnostic delay (mean duration 16 days). Patients presenting acutely to the Emergency Department and patients with neurological deficits were found to have significantly shorter delays. Preoperative neurological deficits (*p* = 0.001) and preoperative red flag symptoms of cord compression (*p* = 0.008) were significant factors that were predictive of postoperative functional independence. Referral delay was also a significant predictive factor (*p* = 0.013); surgical delay approached statistical significance (*p* = 0.075). Conclusions: The results of this study highlight the need for increasing patient education, enhancing physician management of patients with MESCC, and improving diagnostic efficiency to reduce delays and maximize patient outcomes.

## Simple Summary

The spine is the most common site of skeletal metastases. Metastatic epidural spinal cord compression is a time-sensitive oncological emergency, and delays in treatment may lead to irreversible neurological deficits and loss of function. The aim of our retrospective study is to identify the patterns of treatment delay in patients with metastatic epidural spinal cord compression and determine the factors predictive of postoperative ambulatory function. We found that patient delay and diagnostic delay are the most significant factors contributing to overall delay. Preoperative neurological status and delays in referral or surgical treatment were also noted to be predictive of postoperative functional status. These findings identified areas in the chain of care that need to be optimized so as to improve patient outcomes.

## 1. Introduction

The spine is the most common site of skeletal metastases [1,2], and the clinical incidence of spinal metastases is estimated to be approximately 15–30% [3,4,5], with metastatic epidural spinal cord compression (MESCC) occurring in up to 9.6% of patients [6]. Cancers of the breast, lung, and prostate are the most common primary malignancies to metastasize to the spine [7,8,9].

Delays in diagnosis and treatment can potentially result in serious, deleterious effects on patient outcomes and postoperative morbidity [10,11]. In addition, poorer surgical outcomes directly translate to greater burdens of postoperative care, leading to higher healthcare costs incurred by the patient [12]. This is especially so in symptomatic MESCC, a time-sensitive oncological emergency, where treatment delays may lead to irreversible neurological deficits and loss of function.

Expedient diagnosis and treatment are, thus, imperative for improving patient outcomes. The chain of care begins with the recognition of abnormal symptoms by the patient, initial presentation to a healthcare provider, further diagnostic imaging and a referral to a spine surgeon, and, finally, surgical intervention. Delays may occur at any step in this chain of care (i.e. patient delays, diagnostic delays, referral delays, and surgical delays), contributing to poorer postoperative outcomes and increasing the risk of complications that are potentially preventable with early treatment. Van Tol et al. [13] have previously identified the different delay intervals that contributed to the total time to final surgical treatment, but did not directly examine the effect of various clinical factors in predicting postoperative outcome and function, as well as the correlation between these factors and delay intervals. This study aims to analyze patterns of treatment delays and identify predictive factors for postoperative ambulatory function in patients with symptomatic MESCC.

## 2. Materials and Methods

This is a retrospective study conducted at the National University Hospital, Singapore: a tertiary referral center in Singapore. This study was conducted in accordance with the Declaration of Helsinki and approved by the Institutional Review Board, the Domain Specific Review Board, which is under the purview of the National Healthcare Group of Singapore (IRB approval numbers 2020/00495 and 2022/00866; dates of approval: 20 November 2020 and 23 March 2023).

### 2.1. Inclusion and Exclusion Criteria

All adult patients aged 18 years and above who underwent surgical treatment for metastatic epidural spinal cord compression (MESCC) between January 2015 and December 2022 were included in this study. Patients who had primary spine tumors, or who had undergone vertebroplasty, kyphoplasty, or other interventional procedures, were excluded.

### 2.2. Data Parameters

Relevant demographic and clinical data that were extracted included age, sex, and preoperative Eastern Cooperative Oncology Group (ECOG) Performance Status. Oncological data that were collected included primary tumor histology, number of extra-spinal metastases, number of vertebral body metastases, and number of visceral metastases.

Tumors were categorized as slow-growth, moderate-growth, or rapid-growth based on classifications defined by the Skeletal Oncology Research Group [14,15]. Vertebral metastases were identified as metastatic lesions within the vertebral column on whole-spine magnetic resonance imaging (MRI). Extra-spinal bone metastases referred to metastatic skeletal lesions located outside the vertebral column, while visceral metastases referred to lesions in internal organs such as the liver and lungs, detected on oncologic staging scans, e.g., computed tomography of the thorax, abdomen, and pelvis (CT-TAP). Oncological history, including whether the primary tumor was known or newly diagnosed at the time of MESCC treatment, was also documented.

Clinical data collected for this study included the presence of preoperative neurological deficits, the type of surgery (emergency or elective), survival status, survival duration, and postoperative ambulatory status (independent or dependent). Presenting symptoms were classified according to the guidelines from the British National Institute of Health and Care Excellence (NICE) [16]. Symptoms indicative of spinal metastases, necessitating a spinal MRI to be performed within one week, included severe, unremitting, mechanical, and/or progressive back pain, night pain, localized tenderness, and/or claudication. Symptoms and signs suggestive of spinal cord compression, necessitating a spinal MRI to be performed within 24 hours, included bladder or bowel dysfunction, gait disturbances, limb weakness, neurological signs of cauda equina compression, paresthesia or sensory loss, and/or radicular pain.

All cases were managed by a multidisciplinary team comprising a spine surgeon, oncologist, radiation oncologist, and other specialist physicians of consultant grade. Decisions for surgical intervention took into account the patient’s neurological status, severity of cord compression as defined by the epidural spinal cord compression (ESCC) scale or Bilsky score, spinal stability (based on the spinal instability neoplastic score (SINS)), oncological status, and prognosis.

Based on the protocol used by the Global Spine Tumour Study Group [17], surgery was defined as elective if it could be scheduled more than 3 days after presentation to the spine surgery team, in the absence of red flag symptoms or signs. Surgery was classified as non-elective or emergent if performed within three days of presentation to the spine surgery team due to red flag symptoms such as neurological deficits or significant spinal instability.

Patterns of delay in receiving final surgical intervention were categorized into distinct phases of the patient’s care pathway, as described by Van Tol et al. [13]. Patient delay was defined as the time between the onset of symptoms and the patient’s initial presentation to a healthcare provider. Diagnostic delay referred to the time from the patient’s first presentation to the confirmation of spinal metastases diagnosis. Referral delay was the time between diagnosis and referral to the spine surgery team, while surgical delay was the time from referral to the spine surgery team to the final surgical intervention. We conducted a thorough review of the electronic medical records in order to determine the respective delays. Within our institutional healthcare cluster, all medical records are organized into an integrated electronic medical records system. Patients who do not originally reside within the boundaries of the institutional healthcare cluster will also have their clinical data made available to their respective healthcare teams, for the purpose of clinical care, via the national electronic medical records, once they come within our care. We are, thus, able to trace and determine accurately the diagnostic delay (medical consultation to diagnosis), referral delay (diagnosis to spine team referral), and treatment delay (spine team referral to treatment), as these dates are readily available on the electronic medical records. To determine patient delays and the durations of symptoms, we reviewed notes from the primary healthcare provider and Emergency Departments, as well as the oncological and surgical teams; if there is consistency in the reported timings of symptom onset, they were then used for the determination of patient delay. If the timings were inconsistent, the mean of the reported timings was utilized to determine patient delay. Delay durations are expressed in days or weeks; when the patient reports the duration of their symptoms in terms of months, the length of a month was standardized to be 30 days for the purpose of data collection. This standardized approach was adopted to collect all of our data so as to ensure consistency. Where there was doubt with regard to the duration of diagnostic delay, a collective decision was made by the authors through discussion and consensus.

### 2.3. Statistical Analysis

Standard statistical methods used for analysis include the chi-square test of association, Fisher’s exact test (for categorical variables), *t*-test, Mann–Whitney U test, and one-way analysis of variance (ANOVA) (for continuous variables). Statistical significance was set at 5% (*p* = 0.05).

Univariate analysis was used to identify predictive factors for postoperative ambulatory function. Factors that were identified to be statistically significant on univariate analysis were further analyzed on a multivariate logistic regression model.

Patterns of delay were then compared between patients with and without predictors of postoperative ambulatory function.

All data were analyzed using SPSS (version 29; IBM, Armonk, NY, USA).

## 3. Results

### 3.1. Patient Characteristics

A total of 177 patients who underwent surgical treatment for MESCC at our institution were included in this study. Patient characteristics are shown in Table 1.

These patients had a mean age of 62.6 years (standard deviation 10.1 years) and an approximately equal gender ratio (M:F = 1:1.1). The majority of patients had good preoperative functional status, as defined by an ECOG score of 0–2 (93.8%, 166/177). Slow-growth and moderate-growth primary tumors accounted for more than 70% (126/177) of the cases. In total, 39.0% (69/177) of patients did not have any extraspinal metastases; of the patients with vertebral metastases, 62.1% (110/177) had three spinal lesions or more. Visceral metastases were present in 55.9% (99/177) of the patients in our cohort.

Most patients (61.6%, 109/177) had a known diagnosis of cancer prior to their presentation for MESCC; a smaller proportion of patients (38.4%, 68/177) did not have a known malignancy. The numbers of patients with (52.5%, 93/177) or without (47.5%, 84/177) preoperative neurological deficits were approximately equal. Overall, 78.5% (139/177) of patients had red flag symptoms of cord compression. The numbers of elective (51.4%, 91/177) and emergency (48.6%, 86/177) surgeries were approximately equal. The median survival duration for this patient cohort was 13.0 months (range 1.0–92.0 months), with 9 patients (5.1%) lost to follow-up. Postoperatively, 52.0% (92/177) of patients achieved independence in ambulation, while 48.0% (85/177) remained dependent. Among the dependent group, 4.5% (8/177) were ambulant with assistance, 7.9% (14/177) used a walking stick, 16.4% (29/177) relied on a walking frame, 13.0% (23/177) were wheelchair-bound, and 6.2% (11/177) were bedbound.

### 3.2. Predictive Factors of Postoperative Ambulatory Status

On univariate analysis (Table 2), patients who presented to the Emergency Department (vs. elective outpatient clinics), underwent emergency surgery, or had red flag symptoms suggestive of cord compression were significantly less likely to achieve postoperative independence (*p* < 0.05). Patients who did not have preoperative neurological deficits were significantly more likely to achieve postoperative independence (OR = 5.30, 95%CI = 2.78–10.11, *p* < 0.001). Known history of malignancy was not found to be a significant predictor of postoperative independence.

Multivariate analysis revealed that only the absence of preoperative neurological deficits and the presence of red flag symptoms suggestive of cord compression were statistically significant predictors of postoperative ambulatory status. Patients without preoperative neurological deficits were significantly more likely to achieve postoperative independence (adjusted OR = 3.27, 95%CI = 1.58–6.77, *p* = 0.001). Conversely, patients with preoperative red flag symptoms suggestive of cord compression were less likely to achieve postoperative independence (adjusted OR = 0.26, 95%CI = 0.09–0.70, *p* = 0.008).

The impact of other factors such as age, sex, SORG classification of the primary tumor, premorbid ECOG status, and level of surgical intervention on outcomes was also analyzed; however, these factors were not found to be statistically significant on multivariate regression analysis (*p* > 0.05).

### 3.3. Patterns of Delay

**Figure 1:**
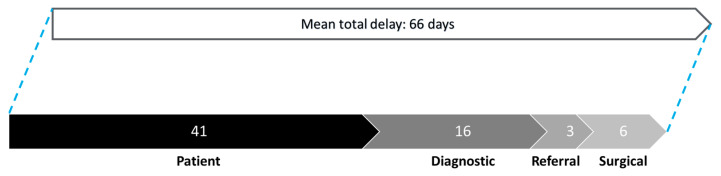
Patterns of delay for overall study cohort.

Figure 1 illustrates the patterns of delay for the overall patient cohort. The mean total delay, from symptom onset to final surgical intervention, was 66 days. The contributing delay factors, in descending order of significance, are patient delay, diagnostic delay, surgical delay, and referral delay. Among the contributing factors, patient delay was the most significant, with a mean duration of 41 days. The mean durations for other delays were diagnostic delay, 16 days; referral delay, 3 days; and surgical delay, 6 days.

Referral delay was identified as a significant predictor of postoperative ambulatory status in multivariate logistic regression analysis (adjusted OR = 1.11, 95%CI = 1.02–1.20, *p* = 0.013). Surgical delay also showed a trend toward statistical significance as a predictive factor (adjusted OR = 1.04, 95%CI = 0.99–1.08, *p* = 0.075) (Table 3).

### 3.4. Subgroup Analysis

**Figure 2:**
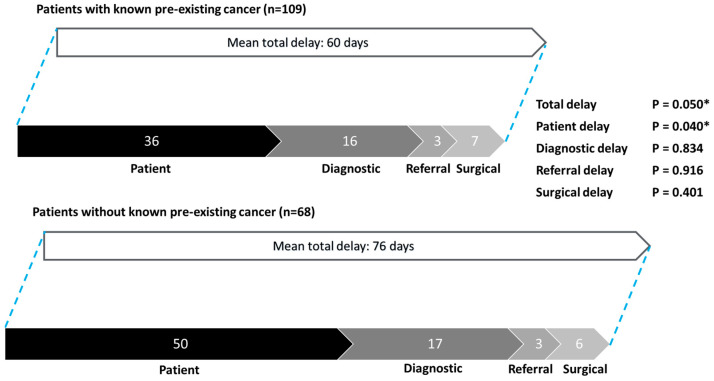
Patterns of delay: patients with known pre-existing cancer vs. patients without known pre-existing cancer. * Statistically significant.

Subgroup analysis showed that total delay was significantly shorter for patients with known cancer as compared to patients without pre-existing malignancies (mean 60 days, vs. 76 days; *p* = 0.050) (Figure 2). Significantly shorter patient delay was also found in patients with known cancer (mean 36 days, vs. 50 days; *p* = 0.040).

**Figure 3:**
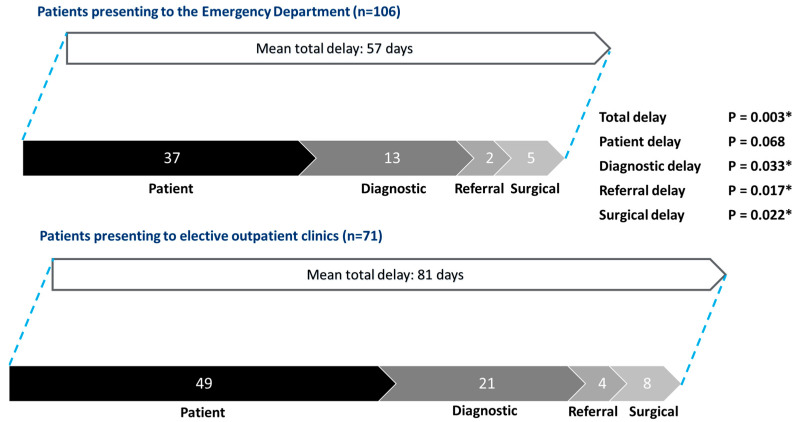
Patterns of delay: patients presenting to the Emergency Department vs. patients presenting to elective outpatient clinics. * Statistically significant.

Patients presenting to the Emergency Department experienced significantly shorter total delay, diagnostic delay, referral delay, and surgical delay compared to those presenting to elective outpatient clinics (Figure 3). The mean total delay was 57 days for Emergency Department presentations versus 81 days for outpatient clinics (*p* = 0.003). The breakdown of delays was as follows: patient delay (37 days vs. 49 days, *p* = 0.068), diagnostic delay (13 days vs. 21 days, *p* = 0.033), referral delay (2 days vs. 4 days, *p* = 0.017), and surgical delay (5 days vs. 8 days, *p* = 0.022).

**Figure 4:**
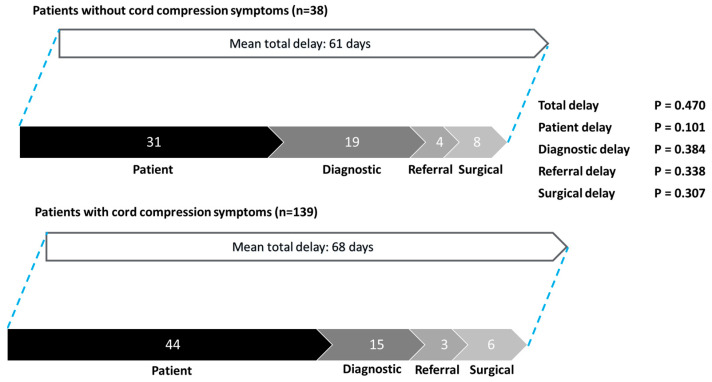
Patterns of delay: patients without cord compression symptoms vs. patients with cord compression symptoms.

Comparing patients without cord compression symptoms to patients with cord compression symptoms, there were no significant differences in terms of total delay (mean 61 days vs. 68 days, *p* = 0.470), patient delay (mean 31 days vs. 44 days, *p* = 0.101), diagnostic delay (mean 19 days vs. 15 days, *p* = 0.384), referral delay (mean 4 days vs. 3 days, *p* = 0.338), or surgical delay (mean 8 days vs. 6 days, *p* = 0.307) (Figure 4).

**Figure 5:**
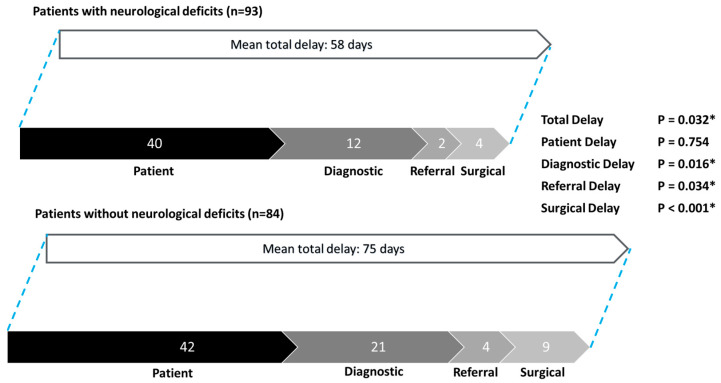
Patterns of delay: patients with neurological deficits vs. patients without neurological deficits. * Statistically significant.

Total delay, diagnostic delay, referral delay, and surgical delay were all significantly shorter for patients with neurological deficits compared to those without (Figure 5). The mean total delay was 58 days for patients with neurological deficits versus 75 days for those without (*p* = 0.032). The breakdown of delays was as follows: patient delay (40 days vs. 42 days, *p* = 0.754), diagnostic delay (12 days vs. 21 days, *p* = 0.016), referral delay (2 days vs. 4 days, *p* = 0.034), and surgical delay (4 days vs. 9 days, *p* < 0.001).

## 4. Discussion

### 4.1. Importance of Early Surgical Treatment of MESCC

The role of surgical intervention in the management of MESCC is established in the literature; in a landmark randomized control trial by Patchell et al. [18], decompressive surgery with adjuvant radiotherapy was shown to be superior to radiotherapy alone for the treatment of MESCC. In addition, current evidence also suggests that certain patient groups, traditionally thought to be more vulnerable to operative risks due to factors such as age, poor premorbid functional status, or limited life expectancy, may also benefit from surgical intervention. Elderly patients (aged 70 years or older) who underwent surgical intervention were found to have postoperative outcomes comparable to those of younger patients [19]. Patients with poorer ECOG status also benefited from surgery, in terms of preservation of neurological function, increased ability to ambulate, and reduction in pain [20]. Finally, patients with poor life expectancy (<3 months) had comparable quality of life to patients surviving > 3 months after undergoing surgery [21]. The clinical utility of surgical intervention is well established beyond doubt. Additionally, growing evidence highlights the importance of early surgery in maximizing patient outcomes, as delays are directly associated with increased morbidity and a higher risk of complications.

Consistent with the current literature, this study’s findings indicate that the prompt surgical management of MESCC is essential for improving patient outcomes and increasing the likelihood of postoperative functional independence. Both referral delay and surgical delay were found to directly correlate with postoperative outcomes, with surgical delay approaching statistical significance. Although patient delay and diagnostic delay did not show statistical significance in predicting postoperative ambulatory status, this may be confounded by data heterogeneity. The likelihood of achieving postoperative functional independence is likely inversely correlated with the extent of irreversible neurovascular injury to the spinal cord. Greater delays in treatment increase the ischemia time of the cord and the risk of permanent neurological damage. Prior research has identified delayed presentation to a spine surgeon as the strongest predictor of poor postoperative outcomes in patients treated for spinal metastases [10]. Similarly, a study by Hsieh et al. [22] examining the impact of COVID-19-lockdown-related treatment delays in Taiwan found that survival probability was highest after the lockdown was lifted and lowest during the lockdown, with increased delays in surgical treatment correlating with higher mortality risk.

This inverse relationship is reflected in the clinical variables identified in this study as significant predictors of postoperative ambulatory status. Indicators such as the presence of red flag symptoms of cord compression, emergent surgery, and preoperative neurological deficits directly reflect the extent of spinal cord compromise. Presentation to the Emergency Department serves as an indirect indicator, as patients with more severe symptoms of worsening spinal cord compromise are more likely to seek urgent medical attention rather than wait for an elective outpatient clinic appointment.

From the results of our study, it is evident that delays in treatment are significant in contributing to poorer patient outcomes. Measures should, thus, be taken to address all causes of delay in the chain of care as described.

### 4.2. Reducing Patient Delay Through Patient Education and Collaboration with Primary Care Providers

Patient delay was the most significant contributor to total delay across the overall cohort and individual subgroups. Notably, patients with known malignancies experienced significantly shorter patient delays and overall delays compared to those without a cancer history. This may reflect greater awareness among patients with oncological histories, who are counselled by their oncologist and surgeon on concerning symptoms, thus prompting them to seek medical attention when experiencing such symptoms. In contrast, patients without pre-existing malignancies in our cohort had patient delays that were up to 40% longer (mean 50 days vs. 36 days), underscoring the need to improve public health awareness.

The initial symptoms of spinal metastases are often non-specific, with back pain being a common but potentially misleading symptom, as it can also result from non-malignant conditions such as degenerative spinal disease. In this study, mean patient delay was significantly longer than in Western populations. For instance, mean patient delay was 19 days for Dutch patients in the study by van Tol et al. [13], and a Scottish study by Levack et al. [23] reported that 83% of patients sought medical attention for back pain within three weeks of symptom onset. Similarly, studies in England and Poland by Husband [24] and Guzik [25], respectively, reported mean delays as short as 3–4 days. The longer delays observed in the local cohort may reflect a lack of health awareness and prevailing cultural attitudes among older patients in Singapore, such as a tendency to dismiss personal medical complaints [26]. These patients are often less proactive in seeking medical care. Educating elderly patients and the general public about the importance of seeking prompt medical attention for persistent symptoms, such as back pain, is crucial. Public health campaigns should focus on raising awareness of red flag symptoms associated with back pain [16], as such symptoms that might otherwise be incorrectly dismissed as merely signs of aging. Greater awareness will enable patients to self-screen for suspicious features and consult a physician without delay, potentially preventing more severe complications.

General practitioners play a critical role in the chain of care as the first point of contact for most patients [27]. There is a pressing need for primary care physicians to foster collaborative and constructive relationships with patients to enhance education and enable the early detection of suspicious symptoms. At-risk individuals, such as those with a known oncological history, could also benefit from closer outpatient specialist follow-up.

### 4.3. Optimizing Multidisciplinary Care of MESCC Patients to Reduce Delays

The findings of this study highlight the importance of optimizing physician management of patients with MESCC to reduce delays and improve outcomes. Patients presenting acutely to the Emergency Department experienced significantly shorter diagnostic, referral, and surgical delays compared to those presenting electively to outpatient clinics. The severity of their symptoms likely prompts faster diagnostic evaluation, referral to a spine surgeon, and surgical intervention. Neurological deficits identified during the initial consultation also resulted in accelerated care, as these deficits signaled clinical urgency to the attending physicians. However, in this cohort, the presence of red flag cord compression symptoms did not significantly reduce delays, emphasizing the need for efficient pathways for the urgent management of MESCC. A multidisciplinary approach involving the surgical, medical oncology, radiation oncology, and radiology teams is essential to fast-track the care of patients with suspected MESCC.

Guidelines by the British National Institute of Health and Care Excellence (NICE) recommend the involvement of specialties such as hematology–oncology, radiology, histopathology, spine surgery, and palliative care in the care of MESCC patients [16]; in addition, a health professional (such as an advanced specialty nurse) should be designated as the point of contact to facilitate the coordination of care for patients. In particular, the guidelines also specified that the role of the MESCC coordinator should be covered 24/7; when the MESCC coordinator is not working, a clinician (such as an on-call registrar) should assume the duties of the coordinator [16]. By ensuring that the MESCC service is readily contactable at all times, potential delays in referral and treatment can be reduced.

Direct lines of communication between relevant specialties should also be established to promote interdisciplinary cooperation. For example, this can be achieved by convening multidisciplinary tumor boards (MTBs) to allow for the discussion of complex cases and facilitate information sharing [28]. MTBs have been shown to improve adherence to clinical guidelines and enhance the survival of oncology patients [29]. A centralized institutional data repository containing the compiled clinical, surgical, radiological, and histopathological data of all MESCC patients may also be set up to support future research and enhance patient outcomes.

### 4.4. Utilizing Artificial Intelligence and Machine Learning Diagnostic Tools to Reduce Diagnostic Delay

This study also underscores that, after patient delay, diagnostic delay is the next most significant contributor to overall delay, aligning with the findings of previous studies [13,23,24,25]. In addition to increasing physician awareness of MESCC and streamlining multidisciplinary care pathways, healthcare providers should consider adopting advanced analytical tools to enhance diagnostic efficiency. With the growing role of artificial intelligence (AI) in healthcare, AI and machine learning hold promise as diagnostic adjuncts for patients with spinal metastases. These tools could augment conventional diagnostic methods, helping to reduce delays and improve patient outcomes.

Epidural metastases are often missed on computed tomography (CT) scans. A retrospective study at our institution [30] analyzed 123 CT scans from 101 patients with epidural spinal cord compression (ESCC), all of whom also underwent spinal MRI within 30 days. While all patients had cord compression visible on MRI, the original radiologists identified epidural spinal cord compression on only 44% of the preceding CT scans. Following a dedicated review, ESCC was diagnosed on 92% of these CT scans. These findings highlight the importance of radiologists being educated on assessing MESCC severity on CT scans, which could increase the opportunistic detection of MESCC even during routine CT-staging scans. Beyond physician expertise, machine learning (ML) and deep learning (DL) models show significant potential as adjunctive tools for diagnosing MESCC. DL models have demonstrated comparable agreement with subspecialist radiologists and clinical specialists for classifying MESCC on MRI scans using the Bilsky score [31].

Further advancements and refinements in DL techniques could lead to the development of diagnostic algorithms or approaches that integrate these models. Such innovations could enhance the efficiency, reliability, and accuracy of current radiological methods for diagnosing MESCC, providing a diagnostic standard comparable to that of subspecialist radiologists. With the exponential rise in computing power and increasing availability of large datasets in healthcare, AI and machine learning are poised to drive revolutionary changes in medicine over the next few decades. Radiomics is a rapidly growing field in radiology that utilizes AI/ML techniques to improve diagnostic efficiency and accuracy in medical imaging. One of the underlying premises of the science of the radiomics is that biomedical images may contain information of disease processes that may not be detected easily by pure visual inspection [32]. Thus, the adoption of AI techniques aims to overcome human fallibility, minimize subjectivity in the interpretation of radiological images, and detect subtle imaging features that may yield vital information to improve diagnostic accuracy. Computer-assisted diagnostic (CAD) systems have already found applications in breast mammography and computed tomography pulmonary imaging [33], and have been used to distinguish between benign and malignant nodules/lesions with varying degrees of success. A systematic review by Eadie et al. [34] found that the incorporation of CAD systems into imaging diagnostics increased the sensitivity of modalities such as mammography, breast ultrasound, and dermatologic imaging. Deep convolutional neural networks (CNNs) have also been developed to detect cerebral aneurysms at a reported rate of 94.2% (98/104) and sensitivity of 70% [35].

Miki et al. [36] described the workflow and protocol for the integration of CAD systems into a routine clinical practice environment to detect cerebral aneurysms on MR angiograms. Firstly, two radiologists independently reviewed an MR angiogram without CAD input to arrive at an initial diagnosis, following which, a web-based CAD was consulted to yield a post-CAD diagnosis. Finally, the team arrived at a final diagnosis through discussion and consensus. Through this protocol, the detection rate of cerebral aneurysms was increased by 9.3% without sacrificing specificity.

This demonstrates the feasibility of integrating CAD and AI techniques into daily clinical practice. A similar protocol could be adopted for the detection of spinal metastatic lesions in high-risk patients. One such possibility is the use of CAD systems for the opportunistic detection of spinal metastases on routine CT oncologic imaging. These tools can rapidly screen for potentially suspicious findings in the spinal column, prompting further dedicated imaging such as spinal MRI when indicated.

CAD systems have not been fully integrated into routine clinical practice yet as several issues remain unresolved. The use of CAD tools may have an adverse impact on diagnostic specificity [34] and may increase the risk of false-positive results; furthermore, radiomic studies often have poor reproducibility due to the lack of standardization and insufficient reporting [32]. Current evidence is also, to a large extent, retrospective in nature [32]; more prospective studies will need to be conducted to truly validate their clinical utility. The further development of advanced AI models and CAD systems is also hampered by the lack of curation of existing datasets, which prevent their utilization on the scale that is necessary for the widespread adoption of CAD techniques [37]. In addition, their adoption will also place significant logistical and infrastructure demands on hospitals due to the requirements of advanced computational software and the hardware that is necessary to support such software [38]. These issues will need to be addressed before AI and CAD systems can be fully integrated into routine clinical practice.

### 4.5. Study Limitations

Our study has a few limitations. Our specific local population may limit the application of the study’s findings to other healthcare systems, and the fact that cultural, socioeconomic, and systemic factors may have a direct or indirect impact on the results. The relatively small sample size of our study also does not permit more detailed quantitative analysis to be performed on our data so as to identify potential time cutoffs for treatment or surgical intervention. From our analysis, however, it is indeed clear that any form of delay, no matter the duration, could potentially have serious, deleterious effects on patient outcomes. In our study, patient delay and diagnostic delay were found to be the most significant contributory factors to overall delay, and they represent areas in the patient’s chain of care that should be further optimized.

## 5. Conclusions

In conclusion, this study identified significant predictive factors for postoperative ambulatory status and analyzed distinct patterns of treatment delay in patients with MESCC. These findings highlight the importance of increasing patient education, enhancing physician management, and improving diagnostic efficiency to minimize delays and maximize patient outcomes.

## Figures and Tables

**Table 1:** Demographic, oncological, and clinical characteristics of patient cohort. ECOG—Eastern Cooperative Oncology Group; SORG—Skeletal Oncology Research Group.

Variable	*n* (%)
Age, years (mean (standard deviation))	62.6 (10.1)
Sex	
Male	85 (48.0%)
Female	92 (52.0%)
ECOG Score	
0–2	166 (93.8%)
3–4	11 (6.2%)
SORG classification of primary tumor	
Slow growth	63 (35.6%)
Moderate growth	63 (35.6%)
Rapid growth	51 (28.8%)
Number of extra-spinal metastases:	
≥3	62 (35.0%)
1–2	45 (25.4%)
0	69 (39.0%)
Number of vertebral body metastases:	
≥3	110 (62.1%)
2	36 (20.3%)
1	30 (16.9%)
Incidence of visceral metastases	99 (55.9%)
Oncological history	
Known cancer	109 (61.6%)
New diagnosis	68 (38.4%)
Preoperative neurological status	
Neurological deficits present	93 (52.5%)
No neurological deficits	84 (47.5%)
Nature of symptoms	
Symptoms suggestive of spinal metastases	38 (21.5%)
Symptoms suggestive of cord compression	139 (78.5%)
Nature of surgery	
Elective	91 (51.4%)
Emergency	86 (48.6%)
Survival status	
Survival duration, months (median (range))	13.0 (1.0–92.0)
Deceased	119 (67.2%)
Alive	49 (27.7%)
Lost to follow-up	9 (5.1%)
Ambulatory status	
Independent	92 (52.0%)
Dependent	85 (48.0%)
Ambulant with assistance	8 (4.5%)
Walking stick	14 (7.9%)
Walking frame	29 (16.4%)
Wheelchair-bound	23 (13.0%)
Bedbound	11 (6.2%)

**Table 2:** Factors predictive of postoperative ambulatory status on univariate and multivariate analysis. AOR—adjusted odds ratio; CI—confidence interval; ED—Emergency Department; OR—odds ratio. * Statistically significant.

Variable	OR (95%CI)	*p*-Value	AOR (95%CI)	*p*-Value
Patient presentation to ED vs. outpatient clinic	0.46 (0.25–0.85)	0.014 *	0.65 (0.32–1.30)	0.219
Known history of cancer	0.94 (0.51–1.72)	0.839	-	-
Emergency vs. elective surgery	0.49 (0.27–0.90)	0.021 *	0.80 (0.39–1.62)	0.528
No preoperative neurological deficits	5.30 (2.78–10.11)	<0.001 *	3.27 (1.58–6.77)	0.001 *
Presence of red flag symptoms suggestive of cord compression	0.14 (0.06–0.36)	<0.001 *	0.26 (0.09–0.70)	0.008 *

**Table 3:** Effects of different types of delay on the likelihood of achieving postoperative independence. CI—confidence interval; OR—odds ratio. * Statistically significant.

Variable	Adjusted OR (95% CI)	*p*-Value
Total delay	1.00 (0.99–1.01)	0.248
Patient delay	1.00 (0.99–1.01)	0.975
Diagnostic delay	1.01 (0.99–1.02)	0.142
Referral delay	1.11 (1.02–1.20)	0.013 *
Surgical delay	1.04 (0.99–1.08)	0.075

## Data Availability

The data are available upon request due to restrictions pertaining to confidentiality or ethical issues. The data presented in this study are available upon request from the corresponding author. The data are not publicly available due to confidentiality and ethical issues.

## Abbreviations

The following abbreviations are used in this manuscript:

AI	Artificial intelligence
ANOVA	Analysis of variance
CI	Confidence interval
COVID-19	Coronavirus disease
CT	Computed tomography
CT-TAP	Computed tomography scan of the thorax, abdomen, and pelvis
ECOG	Eastern Cooperative Oncology Group
MRI	Magnetic resonance imaging
MESCC	Metastatic epidural spinal cord compression
NICE	National Institute of Health and Care Excellence (of the United Kingdom)
OR	Odds ratio
SINS	Spinal instability neoplastic score
SORG	Skeletal Oncology Research Group
